# Supplementary material for: Superresolution architecture of cornerstone focal adhesions in human pluripotent stem cells
Source: Nat Commun. 2019 Oct 18;10:4756. doi: 10.1038/s41467-019-12611-w (PMC6802214; doi:10.1038/s41467-019-12611-w)
Supplement: Supplementary file 1 — Supplementary Information [file 41467_2019_12611_MOESM1_ESM.pdf]

Supplementary Information

Superresolution architecture of cornerstone focal adhesions in human pluripotent stem cells

Stubb et al.

Supplementary Figures

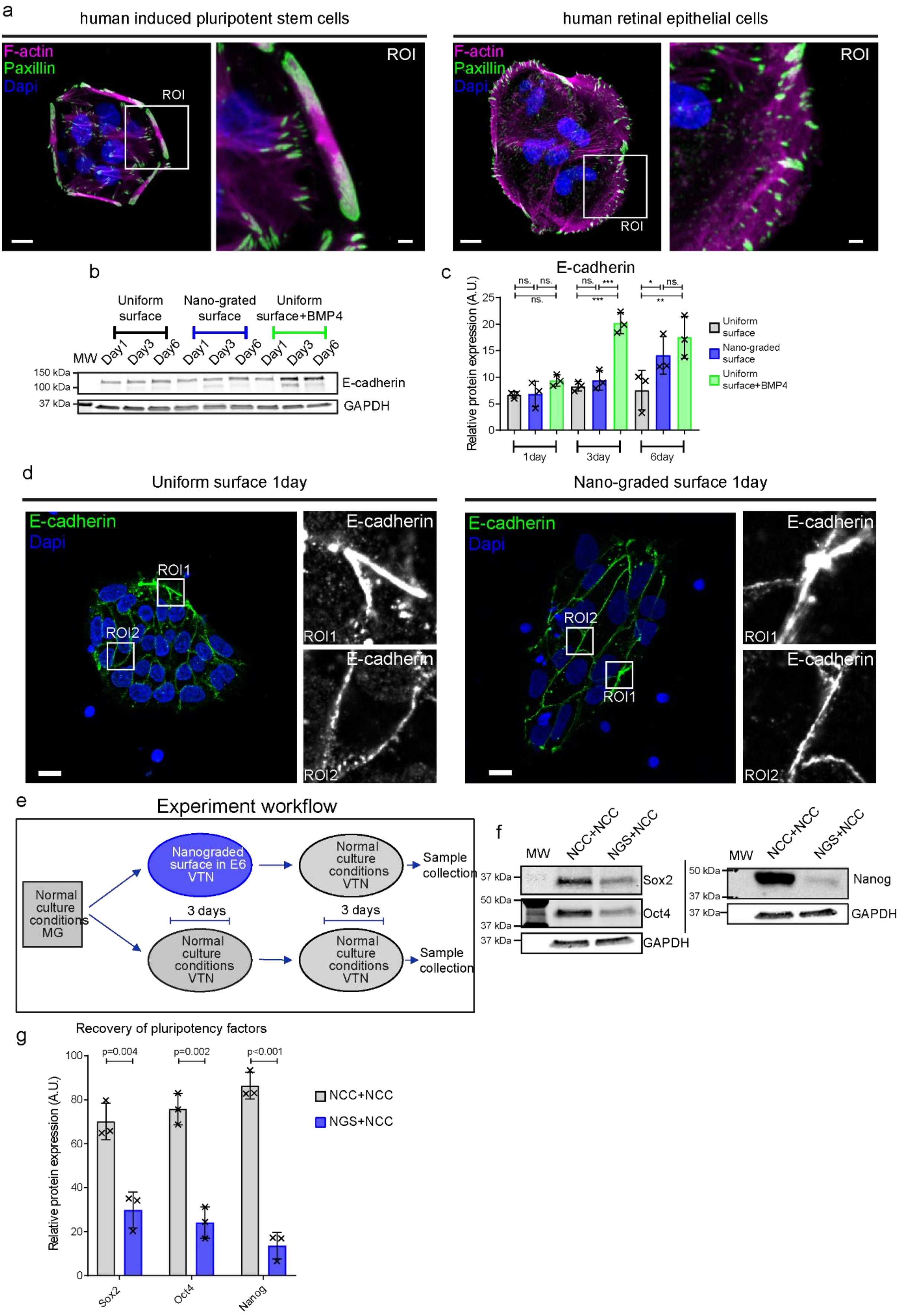

Supplementary Figure 1: hPSC behaviour on nano-grated surfaces

(a) Spinning disk images of hPSC and human retinal epithelial cells (ARPE-19) plated on VTN and stained for F-actin, paxillin, and DAPI. Brightness and contrast were adjusted to allow the best visualization of these structures. Scale bar 10 µm. (b-c) Representative western blot (b) and quantification (c) of E-cadherin levels in hPSC plated for one, three or six days either on VTN-coated uniform surfaces (black/gray), in the presence (green) or absence of BMP4, or on VTN-coated nano-grids (blue) in basal E6 medium (n = 3 biologically independent experiments). Statistics: Oneway-ANOVA with multiple comparisons complemented with Bonferonni's post hoc test. The GAPDH blot presented here is the same one as the one shown in figure 2d. E-cadherin blot presented here was performed on the same membrane as the Sox2 and Oct4 blots presented in figure 2d.

(d) hPSC plated for 24 h on a VTN-coated uniform surface or on a VTN-coated nano-grated surface in basal E6 medium culture conditions, were stained for E-cadherin and DAPI. Images were acquired using a Spinning disk confocal microscope. Scale bars 20 µm. A region of interest 1(ROI1) is magnification from colony edge and region of interest 2 (ROI2) from colony center in each image (n=3 independent experiments).

(e-g) After being plated for three days on a VTN-coated uniform surface (NCC, normal culture condition) or on a VTN-coated nano-grated surface (NGS, nanograted surface), hPSC were replated for three days on a VTN-coated uniform surfaces (e) and the levels of the pluripotency factors Sox2, Oct4 and Nanog were analysed by western blots (n=3 biologically independent experiments) (f,g). Statistics: multiple t-tests (two-tailed, unpaired) with correction using Holm-Sidak method. Error bars depict standard deviation.

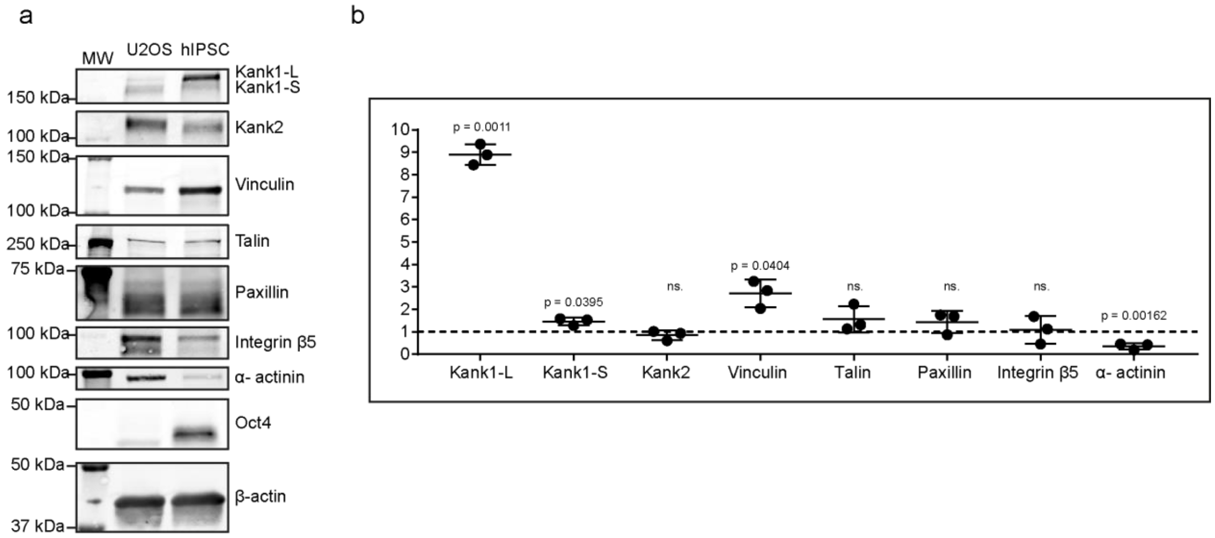

Supplementary Figure 2: Adhesion protein levels in hPSC cells

(a,b) Western blot analysis (a) and quantification (b) of endogenous FA components in U2OS cells and in hPSCs grown on VTN for 24h. Oct4 was used as a pluripotency marker and β-actin as a loading control. For quantification, integrated density values for each protein were first normalised to the loading control for all samples. Results from hPSC were then displayed normalised to U2OS cells (dotted line) (n = 3 biologically independent experiments). Statistics: One sample t-test (two-tailed, unpaired). Error bars depict standard deviation.

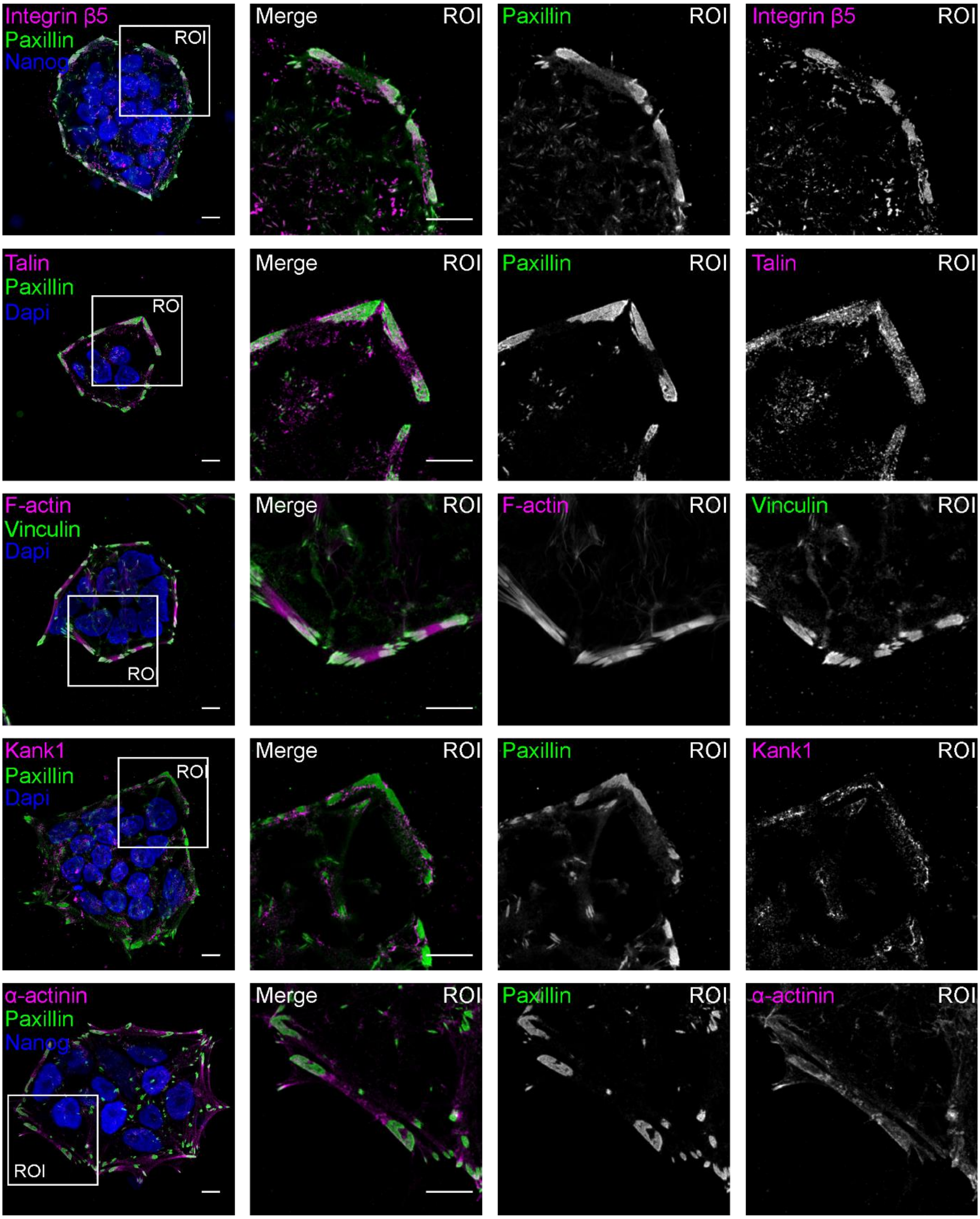

Supplementary Figure 3: Endogenous straining of multiple adhesion proteins in hPSC

Spinning disk images of hPSC plated on VTN and stained for paxillin, vinculin, β5 integrin, talin, kank1, α-actinin, Nanog and DAPI. White squares highlight regions of interests (ROI), which are magnified. Scale bar 10 μm.

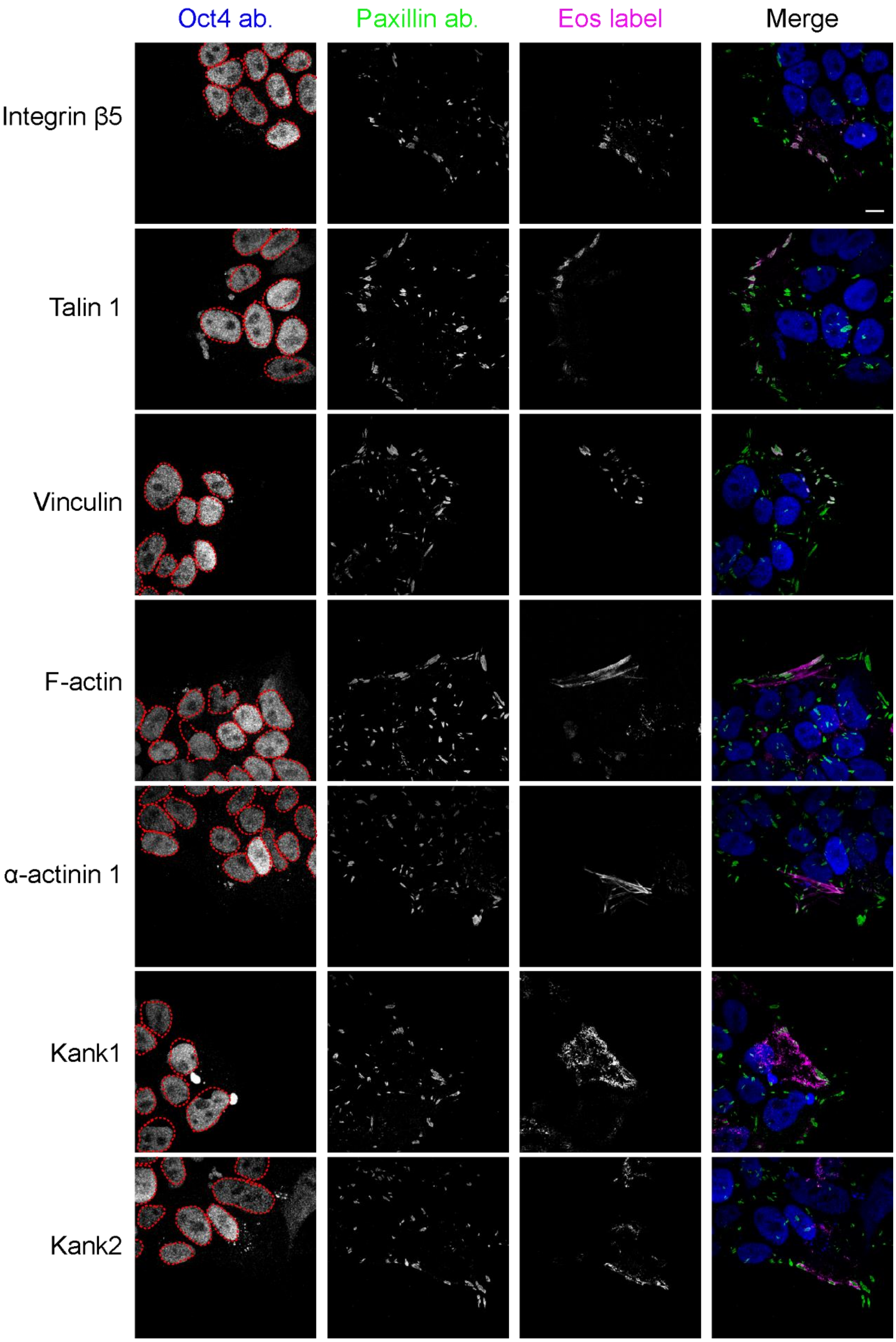

Oct4 ab.
Paxillin ab.
Eos label
Merge
Integrin β5
Talin 1
Vinculin
F-actin
α-actinin 1
Kank1
Kank2

Supplementary Figure 4: Validation of the constructs used for iPALM

Spinning disk images of hPSC plated on VTN and transiently expressing various Eos-constructs used for iPALM in this study. Red dotted lines highlight the nucleus. Oct4 and paxillin were used as pluripotency and FA markers, respectively. Scale bar 10µm.

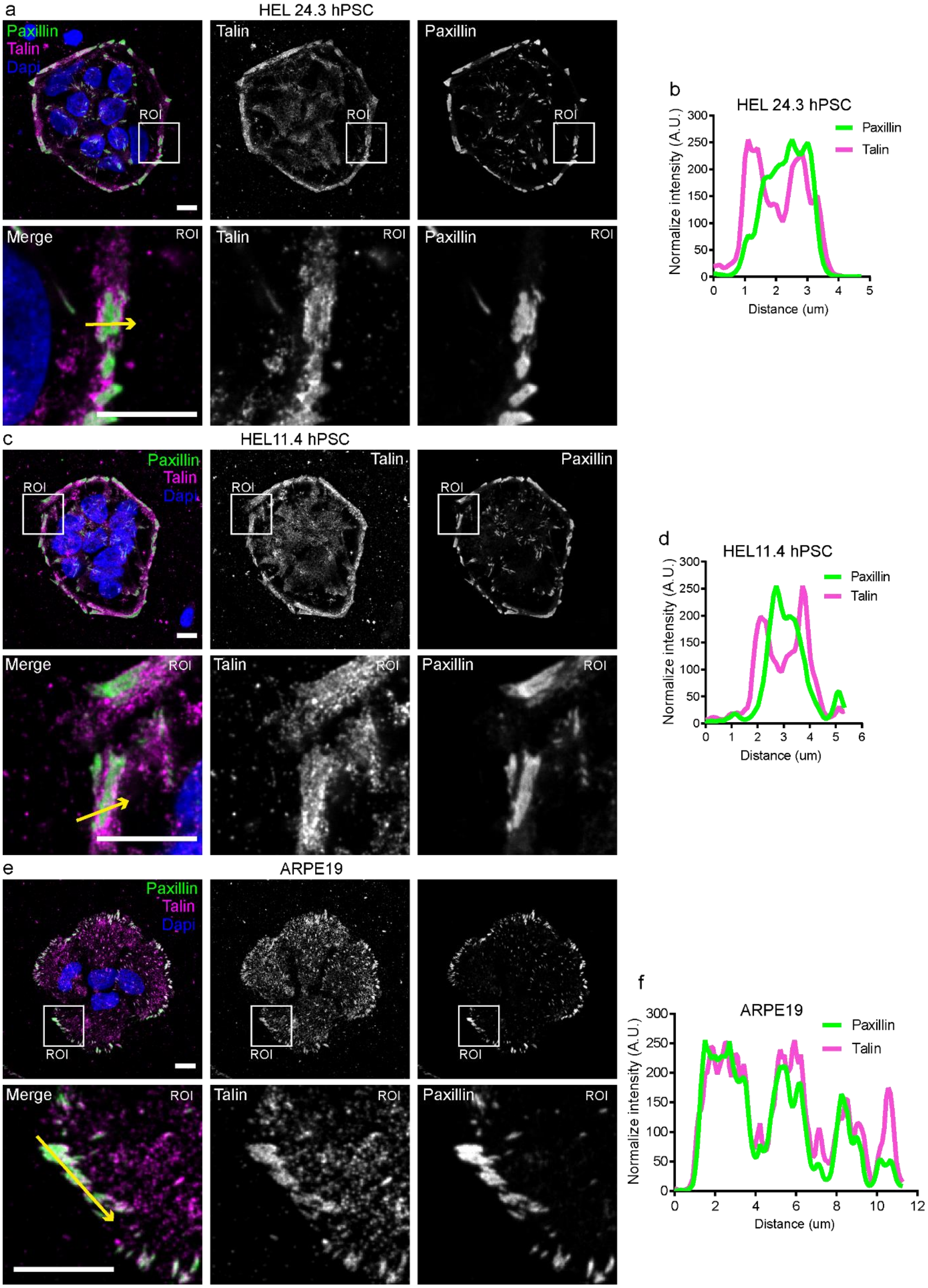

Supplementary Figure 5: lateral segregation of Talin within cornerstone adhesions

(a,c,e) Spinning disk images of two genetically different hPSC lines (a,c) (HEL 24.3 and HEL 11.4) and ARPE-19 cells (e) plated on VTN and stained for endogenous talin and paxillin.

Brightness and contrast adjusted to allow the best visualization of these structures. Scale bar 10 µm. The yellow line in the magnified ROI indicates the area used to measure the intensity profiles displayed in (b,d and f).

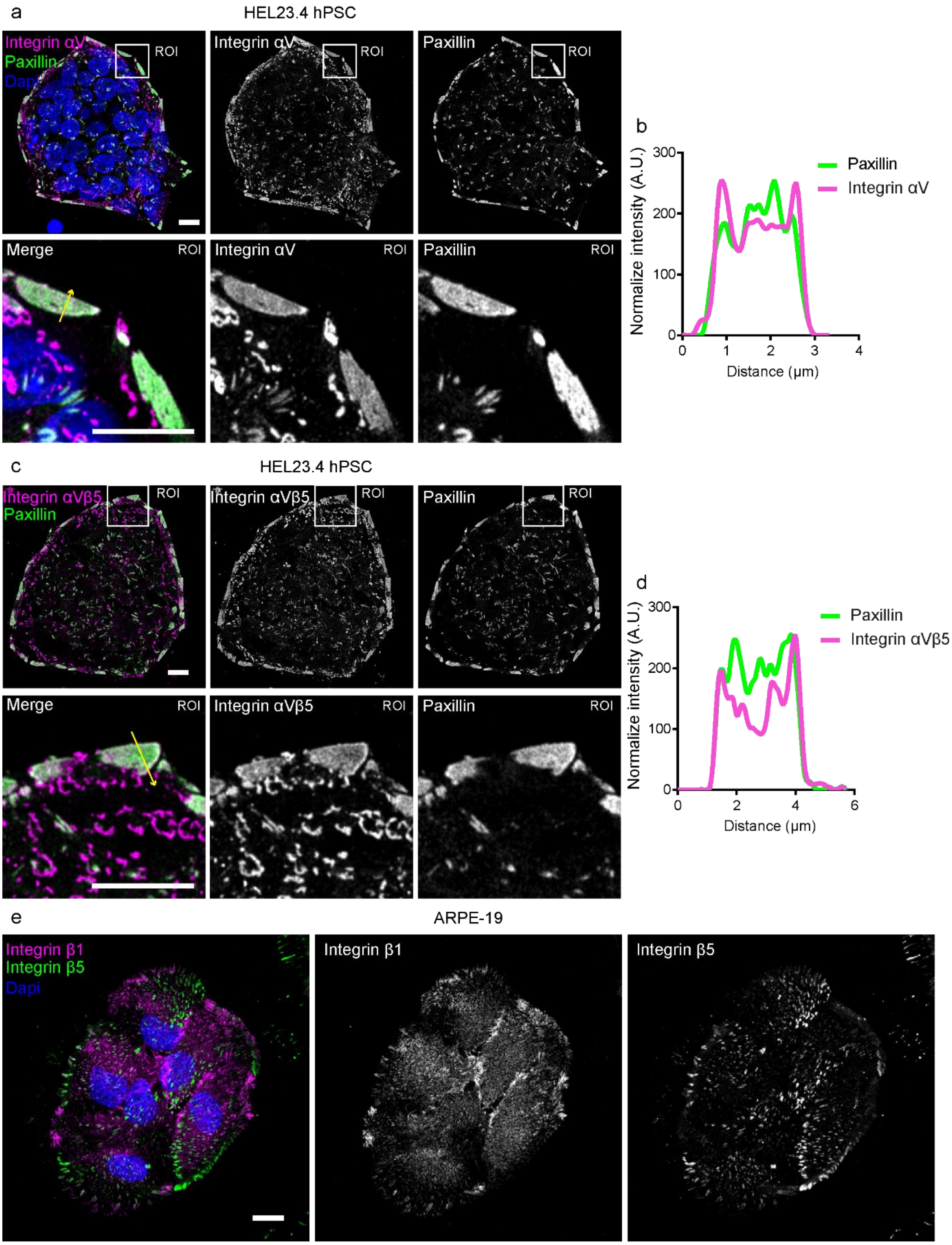

Supplementary Figure 6: lateral segregation of αV and αVβ5 integrin within cornerstone adhesions

(a,c) Airyscan images of hPSC plated on VTN and stained for endogenous αV integrin (a) or αVβ5 integrin (c), paxillin and dapi. Scale bar 10 μm. The yellow line in the magnified ROI indicates the area used to measure the intensity profiles displayed in (b and d). (e) Spinning

disk images of ARPE-19 cells plated on VTN and stained for endogenous β1 integrin, β5 integrin and dapi. Scale bar 10 µm.

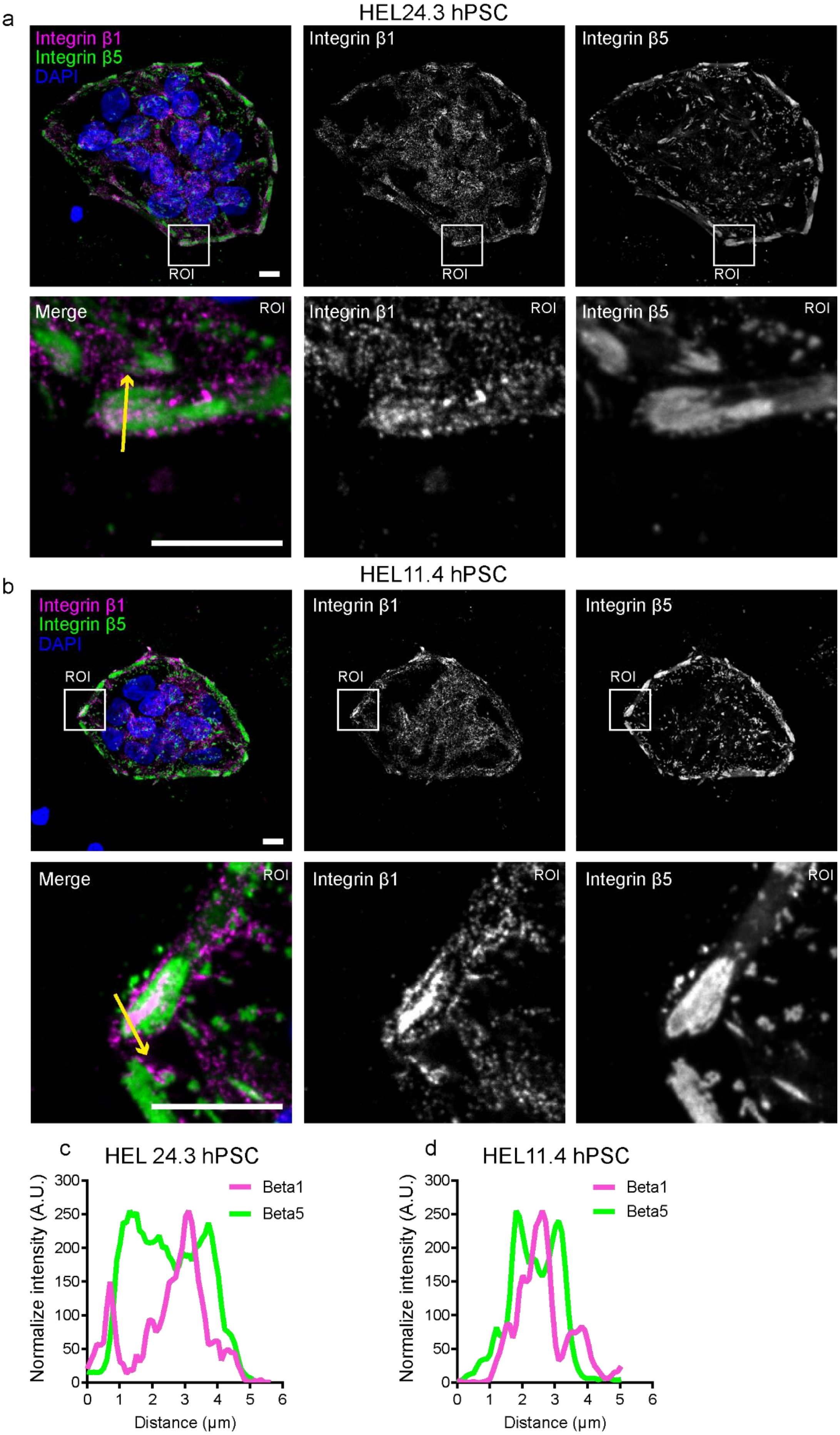
a
HEL24.3 hPSC
Integrin β1
Integrin β5
DAPI
ROI
Merge
b
HEL11.4 hPSC
c
HEL 24.3 hPSC
d
HEL11.4 hPSC
Beta1
Beta5
Normalize intensity (A.U.)
Distance (µm)

Supplementary Figure 7: lateral segregation of β5 integrin within cornerstone adhesions

(a,b) Spinning disk images of two genetically different hPSC lines (HEL 24.3 and HEL 11.4) plated on VTN and stained for endogenous β5 integrin, paxillin and dapi. Scale bar 10 µm. The yellow line in the magnified ROI indicates the area used to measure the intensity profiles displayed in (c and d).

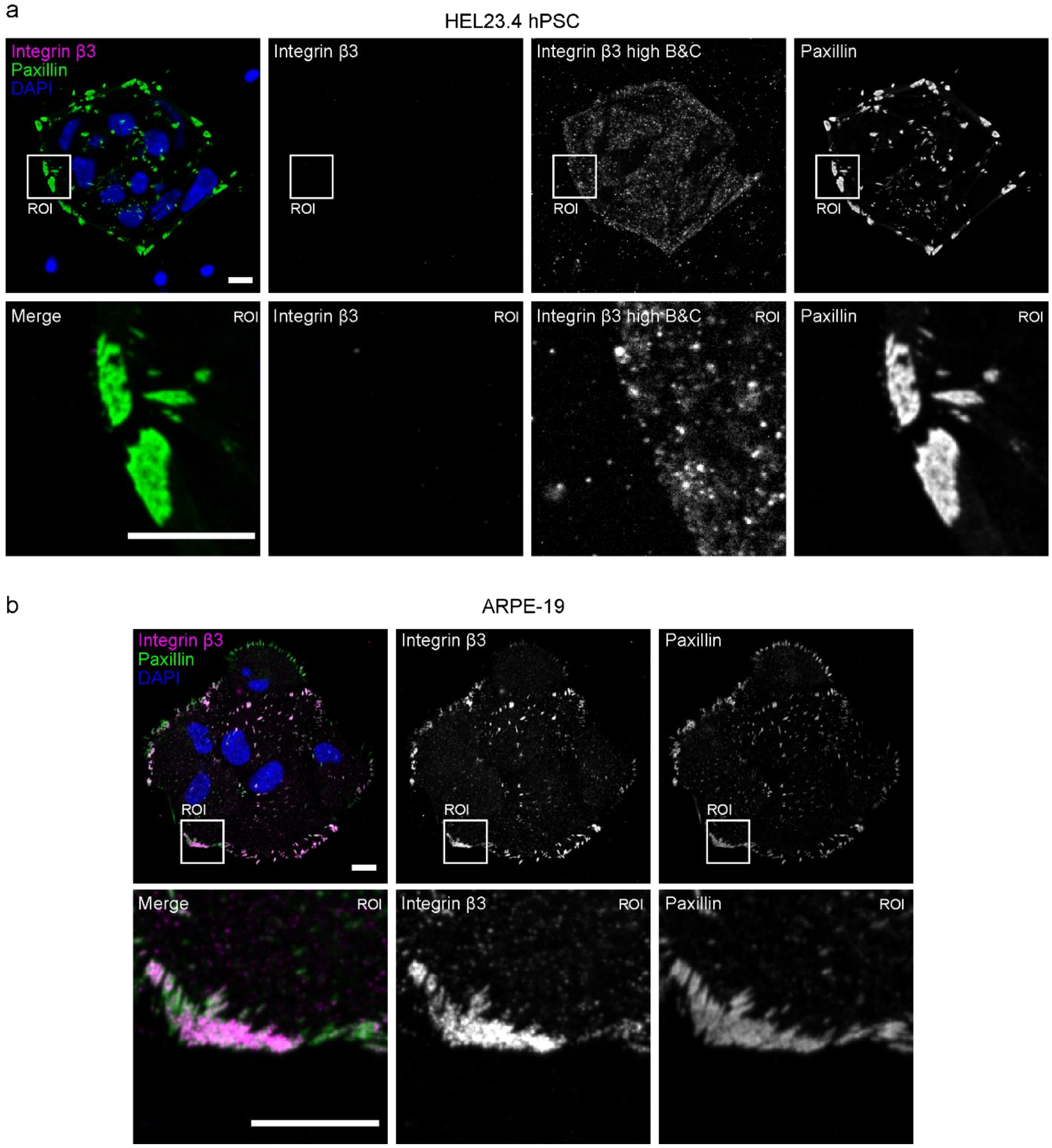

Supplementary Figure 8: β3 integrin levels within cornerstone adhesions

(a,b) Spinning disk images of hPSC or ARPE-19 cells plated on VTN and stained for endogenous β3 integrin, paxillin and dapi. Scale bar 10 µm. Images of the β3 integrin staining in hPSC are displayed using two different contrast settings highlighting the very low detection levels of β3 integrin staining in these cells compared to ARPE-19 cells.

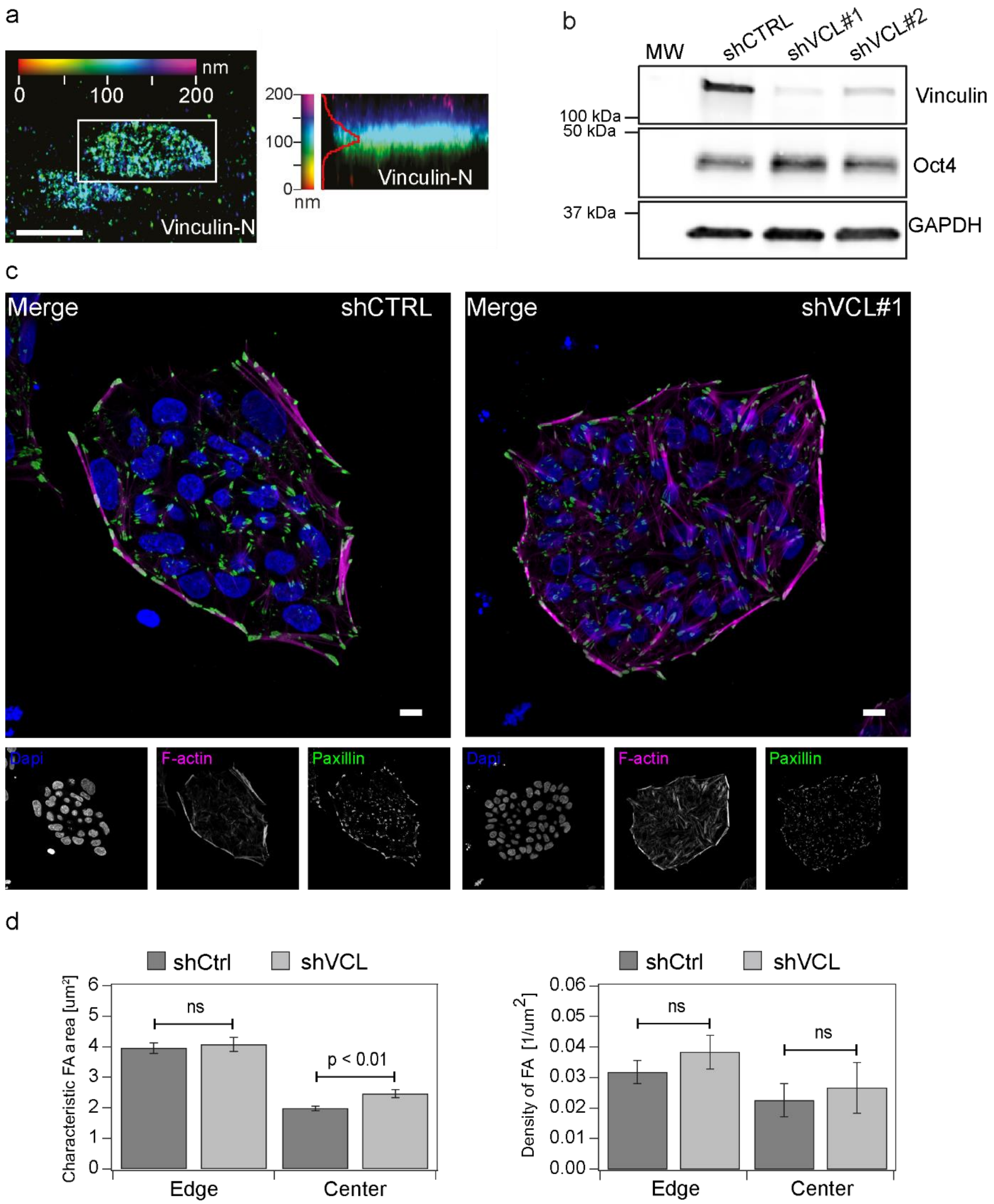

Supplementary Figure 9: Vinculin and cornerstone FA

(a) iPALM images of N-terminally tagged (Eos) vinculin (vinculin-N) in cornerstone FA. Vinculin-N localisation is shown in top-view and side-view images that are colour-coded as a function of the z-position of Vinculin-N molecules. Scale bar 1 μm. **(b)** Vinculin, Oct4 and GAPDH protein levels in hPSC previously infected with lentivirus containing control shRNA (shCTRL) or two individual shRNA targeting vinculin (shVin#1 and shVin#2). **(c)** Spinning disk images of shCTRL or shVinculin hPSC plated on VTN and stained for paxillin, F-actin and DAPI. Scale bar 10 μm. **(d)** Quantification of the characteristic FA area (left) and FA

density (right) at the edge and at the centre of shCTRL and shVinculin hPSC colonies (n = 3 biologically independent experiments). Left: Bars represent the characteristic FA size obtained by fitting a weighted sum of two exponential densities to a histogram of FA area distribution. Error bars depict one standard deviation error in the fit. Right: Bars represent the average density of FAs from the 3 repeats. Error bars correspond to the standard deviation. Statistics: Student t-test (two tailed, unpaired).

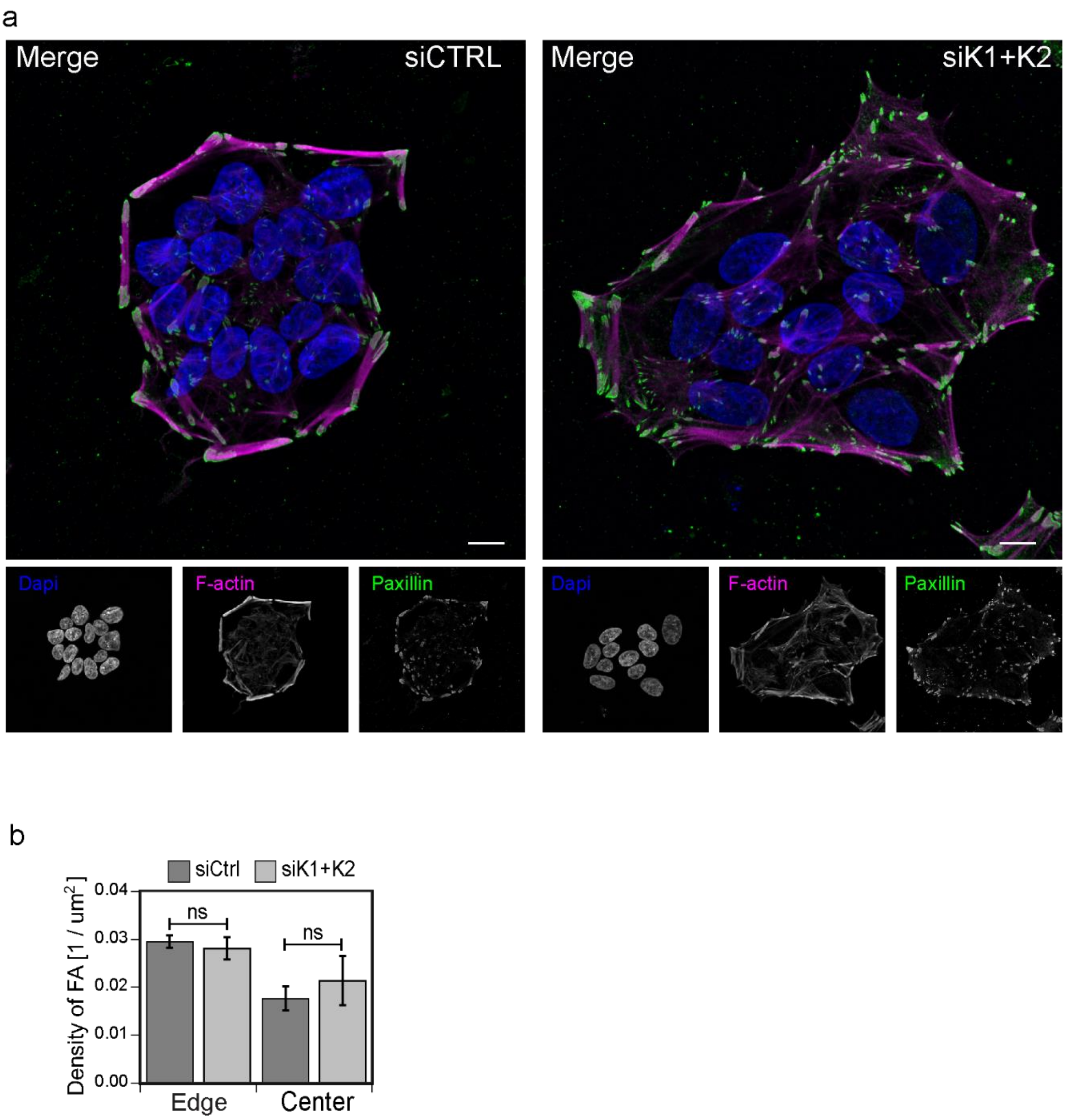

Supplementary Figure 10: kank1 and kank2 in cornerstone FA

(a) Spinning disk images of siCTRL or sikank1 and sikank2 (siK1 + siK2) hPSC plated on VTN and stained for paxillin, F-actin and DAPI. Scale bar 10 µm. **(b)** Quantification of FA density at the edge and at the centre of siCTRL or sikank1 and sikank2 hPSC colonies (n = 3 biologically independent experiments. Bars represent the average density of FAs from the 3 repeats. Error bars correspond to the standard deviation. Statistics: Student t-test (two tailed, unpaired).

Supplementary Tables

Protein	Average Z_{Centre}± S.D. (nm)	# of localisations	# of regions of interest	# of colonies
Integrin αV	45.3 ± 7.6	2.15E+05	17	6
Integrin β5	46.5 ± 10.2	4.78E+05	22	5
Paxillin	55.7 ± 8.5	5.12E+05	23	7
Talin-1-N	72.8 ± 12.3	1.73E+06	54	11
Talin-1-C	103.5 ± 20.5	2.03E+05	8	3
Vinculin-N	100.4 ± 14.4	1.88E+06	55	7
Vinculin-C	76.4 ± 16.3	3.96E+05	27	5
Actin-Low	99.6 ± 14.4	1.11E+06	28	11
Actin-High	155.4 ± 14.2	2.51E+06	47	
α-Actinin-1-Low	125.5 ± 15.1	3.95E+05	27	6
α-Actinin-1-High	174.8 ± 17.1	6.08E+05	31	
Kank-1 Adjacent	116.1 ± 12.1	2.17E+06	29	6
Kank-1 Distal	63.4 ± 13.6	7.08E+05	17	
Kank-2 Adjacent	100.8 ± 13.4	1.91E+06	17	3
Kank-2 Distal	74.2 ± 17.7	4.22E+05	15	

Supplementary Table 1

Table containing the Z_{centre} values (averaged distance from the glass coverslip) of all the proteins imaged in this study using iPALM. This table also contains the number of molecule localisations used to calculate these Z_{centre}, as well as the number of regions of interest and the number of hPSC colonies imaged.
