# Supplementary material for: Superresolution architecture of cornerstone focal adhesions in human pluripotent stem cells
Source: Nat Commun. 2019 Oct 18;10:4756. doi: 10.1038/s41467-019-12611-w (PMC6802214; doi:10.1038/s41467-019-12611-w)
Supplement: Supplementary file 3 — Additional Supplementary Files [file 41467_2019_12611_MOESM3_ESM.pdf]

Description of Additional Supplementary Files

File name: Supplementary Movie 1

Description: Paxillin dynamics in an hPSC colony plated on vitronectin. Live-cell imaging of endogenously tagged paxillin hPSC. Images were acquired using a spinning disk microscope.

File name: Supplementary Movie 2

Description: Nanoscale kank1 localisation in cornerstone adhesions. 3D reconstruction of kank1 (green) and paxillin (red) iPALM data highlighting that kank1 assembles a wall surrounding paxillin-positive adhesions.
