# Supplementary material for: Superresolution architecture of cornerstone focal adhesions in human pluripotent stem cells
Source: Nat Commun. 2019 Oct 18;10:4756. doi: 10.1038/s41467-019-12611-w (PMC6802214; doi:10.1038/s41467-019-12611-w)
Supplement: Supplementary file 6 — Reporting Summary [file 41467_2019_12611_MOESM6_ESM.pdf]

nature research

Corresponding author(s): Johanna Ivaska

Last updated by author(s): 29/8/19

Reporting Summary

Nature Research wishes to improve the reproducibility of the work that we publish. This form provides structure for consistency and transparency in reporting. For further information on Nature Research policies, see Authors & Referees and the Editorial Policy Checklist.

Statistics

For all statistical analyses, confirm that the following items are present in the figure legend, table legend, main text, or Methods section.

n/a	Confirmed	
☐	☒	The exact sample size (*n*) for each experimental group/condition, given as a discrete number and unit of measurement
☒	☐	A statement on whether measurements were taken from distinct samples or whether the same sample was measured repeatedly
☐	☒	The statistical test(s) used AND whether they are one- or two-sided<br>*Only common tests should be described solely by name; describe more complex techniques in the Methods section.*
☒	☐	A description of all covariates tested
☐	☒	A description of any assumptions or corrections, such as tests of normality and adjustment for multiple comparisons
☒	☐	A full description of the statistical parameters including central tendency (e.g. means) or other basic estimates (e.g. regression coefficient) AND variation (e.g. standard deviation) or associated estimates of uncertainty (e.g. confidence intervals)
☒	☐	For null hypothesis testing, the test statistic (e.g. *F*, *t*, *r*) with confidence intervals, effect sizes, degrees of freedom and *P* value noted<br>*Give P values as exact values whenever suitable.*
☒	☐	For Bayesian analysis, information on the choice of priors and Markov chain Monte Carlo settings
☒	☐	For hierarchical and complex designs, identification of the appropriate level for tests and full reporting of outcomes
☒	☐	Estimates of effect sizes (e.g. Cohen's *d*, Pearson's *r*), indicating how they were calculated

Our web collection on statistics for biologists contains articles on many of the points above.

Software and code

Policy information about availability of computer code

Data collection	Data collected to build this manuscript are predominantly microscopy data. These data were collected using commercial software that are associated with the microscopes used (as detailed in the material and methods).
Data analysis	IPALM image analysis was performed using the PeakSelector software (Janelia Research Campus) as previously described previously (Kanchanawong et al., 2010; Shtengel et al., 2009). All other image analyses were performed using ImageJ, freely available at http://imagej.net/Welcome

For manuscripts utilizing custom algorithms or software that are central to the research but not yet described in published literature, software must be made available to editors/reviewers. We strongly encourage code deposition in a community repository (e.g. GitHub). See the Nature Research guidelines for submitting code & software for further information.

Data

Policy information about availability of data

All manuscripts must include a data availability statement. This statement should provide the following information, where applicable:

- Accession codes, unique identifiers, or web links for publicly available datasets
- A list of figures that have associated raw data
- A description of any restrictions on data availability

All data supporting the findings of this study are available within the paper and its supplementary information files, source data file, or from the corresponding author on reasonable request.

Field-specific reporting

Please select the one below that is the best fit for your research. If you are not sure, read the appropriate sections before making your selection.

☒ Life sciences ☐ Behavioural & social sciences ☐ Ecological, evolutionary & environmental sciences

For a reference copy of the document with all sections, see nature.com/documents/nr-reporting-summary-flat.pdf

Life sciences study design

All studies must disclose on these points even when the disclosure is negative.

Sample size	We did not do predetermination of samples size. The number of samples/experiments is indicated separately for each data panel in the figure legends
Data exclusions	Images of poor quality were excluded from analyses. No other data were excluded from the analyses.
Replication	All the experiments included in the manuscript have been reproducible.
Randomization	Experimental groups were defined by experimental conditions. Within an experimental conditions, the allocation was random
Blinding	For the descriptive (IPALM data) no blinding was performed. For the quantitative microscopy data (ie quantification of focal adhesion properties), the imaging and the data analyses were performed by different persons.

Reporting for specific materials, systems and methods

We require information from authors about some types of materials, experimental systems and methods used in many studies. Here, indicate whether each material, system or method listed is relevant to your study. If you are not sure if a list item applies to your research, read the appropriate section before selecting a response.

Materials & experimental systems

n/a	Involved in the study	
☐	☒	Antibodies
☐	☒	Eukaryotic cell lines
☒	☐	Palaeontology
☒	☐	Animals and other organisms
☒	☐	Human research participants
☒	☐	Clinical data

Methods

n/a	Involved in the study	
☒	☐	ChIP-seq
☒	☐	Flow cytometry
☒	☐	MRI-based neuroimaging

Antibodies

Antibodies used	Indicated in the materials and methods section of the manuscript
Validation	All the antibodies have been validated for specificity in our laboratory or validation is provided on the manufacturer's website

Eukaryotic cell lines

Policy information about cell lines

Cell line source(s)	Indicated in the methods section of the manuscript
Authentication	The cells lines were not authenticated
Mycoplasma contamination	All cell lines used were tested for mycoplasma.
Commonly misidentified lines (See ICLAC register)	N/A
